# ORAI1 and ORAI3 in Breast Cancer Molecular Subtypes and the Identification of ORAI3 as a Hypoxia Sensitive Gene and a Regulator of Hypoxia Responses

**DOI:** 10.3390/cancers11020208

**Published:** 2019-02-11

**Authors:** Iman Azimi, Michael J. G. Milevskiy, Silke B. Chalmers, Kunsala T. D. S. Yapa, Mélanie Robitaille, Christopher Henry, Gregory J. Baillie, Erik W. Thompson, Sarah J. Roberts-Thomson, Gregory R. Monteith

**Affiliations:** 1 School of Pharmacy, The University of Queensland, Brisbane 4102, Queensland, Australia; iman.azimi@utas.edu.au (I.A.); s.chalmers1@uq.edu.au (S.B.C.); ktyapa@gmail.com (K.T.D.S.Y.); m.robitaille@uq.edu.au (M.R.); christopher.henry@uqconnect.edu.au (C.H.); sarahrt@uq.edu.au (S.J.R.-T.); 2 Mater Research Institute, Translational Research Institute, The University of Queensland, Brisbane 4102, Queensland, Australia; 3 Division of Pharmacy, College of Health and Medicine, University of Tasmania, Hobart 7001, Tasmania, Australia; 4 ACRF Stem Cells and Cancer Division, The Walter and Eliza Hall Institute of Medical Research, Parkville 3052, Victoria, Australia; milevskiy.m@wehi.edu.au; 5 Division of Genomics, Development and Disease, Institute for Molecular Bioscience, The University of Queensland, Brisbane 4072, Queensland, Australia; gregonomic@gmail.com; 6 Institute of Health and Biomedical Innovation, School of Biomedical Sciences, Translational Research Institute, Queensland University of Technology, Brisbane 4102, Queensland, Australia; e2.thompson@qut.edu.au; 7 University of Melbourne, Department of Surgery, St. Vincent’s Hospital, Melbourne 3065, Victoria, Australia

**Keywords:** ORAI3, ORAI1, Calcium signaling, hypoxia, breast cancer

## Abstract

The remodeling of specific calcium-permeable ion channels is a feature of some breast cancer subtypes. ORAI1 is a protein that forms a calcium-permeable ion channel responsible for store-operated calcium entry (SOCE) in a variety of cell types. ORAI3, a related isoform, is not a regulator of SOCE in most cell types. However, ORAI3 does control SOCE in many estrogen receptor-positive breast cancer cell lines, where it also controls proliferation. ORAI1 is a well-characterized regulator of the proliferation and migration of many basal breast cancer cells; however, the role of ORAI3 in these types of breast cancer cells remains unclear. Here, we sought to define *ORAI1* and *ORAI3* expression in breast cancer cell lines of different molecular subtypes and assess the potential role and regulation of ORAI3 in basal breast cancer cells. Our study demonstrates that elevated *ORAI1* is a feature of basal-like breast cancers, while elevated *ORAI3* is a feature of luminal breast cancers. Intriguingly, we found that *ORAI3* is over-expressed in the mesenchymal subtype of triple-negative breast cancer. Given this, we assessed *ORAI3* levels in the presence of two inducers of the mesenchymal phenotype, hypoxia and epidermal growth factor (EGF). Hypoxia induced *ORAI3* levels in basal breast cancer cell lines through a pathway involving hypoxia-inducible factor-1 alpha (HIF1α). The silencing of ORAI3 attenuated hypoxia-associated phosphorylation of the EGF receptor (EGFR) and the expression of genes associated with cell migration and inflammatory/immune responses in the MDA-MB-468 model of basal breast cancer. Although elevated *ORAI3* levels were not associated with survival; basal, estrogen receptor-negative and triple-negative breast cancers with high *ORAI3* and low *ORAI1* levels were associated with poorer clinical outcomes. This study defines ORAI3 as a potential fine-tuner for processes relevant to the progression of basal breast cancers.

## 1. Introduction

The remodeling of calcium (Ca^2+^) signaling and/or changes in the expression of specific Ca^2+^-permeable ion channels occurs in a variety of cancers [1,2]. In some cases, changes in Ca^2+^ signaling, such as the molecular components of store-operated Ca^2+^ entry (SOCE) are a feature of specific breast cancer subtypes [3]. Breast cancer is a heterogeneous disease with several molecular subtypes including luminal A, luminal B, Her-2 enriched, claudin-low, and basal-like. The vast majority of luminal A and B tumors express the estrogen receptor [4,5]; in contrast, most basal breast cancers are triple negative breast cancers (TNBC) that lack expression of the estrogen, progesterone and ERBB2 (HER2) receptors [4,5]. No targeted therapies for TNBC currently exist, making target identification a major priority to improve the prognosis of this disease [6]. Recently, molecular subtypes of TNBC have been defined and include Basal-Like Immune-Suppressed (BLIS), Basal-Like Immune-Activated (BLIA), Luminal-Androgen Receptor (LAR) and Mesenchymal (MES), which may aid our understanding of how to therapeutically target specific TNBCs [7,8]. TNBC is the most heterogeneous classification of breast cancers, and it remains unknown if there are differences in the Ca^2+^ entry pathways within TNBCs.

The canonical components of SOCE seem to be particularly important in breast cancers of the basal molecular subtype. SOCE is initiated by the depletion of endoplasmic reticulum Ca^2+^ stores, which is detected by the endoplasmic reticulum Ca^2+^ sensor STIM1, resulting in STIM1 mediated activation of plasmalemmal hexameric ORAI1 channels and subsequent Ca^2+^ influx [9,10]. Assessment of microarray datasets indicates that ORAI1 levels are higher in basal breast cancers compared to non-basal [11]. Moreover, silencing and pharmacological inhibition of ORAI1 in basal/triple negative MDA-MB-231 breast cancer cells reduces their invasiveness in vitro and in vivo [12]. However, we still do not have a full understanding of the nature of ORAI1 overexpression in breast cancer molecular subtypes nor the possible drivers for altered expression, such as is evident in the overexpression of TRPV6 in ERα-negative breast cancers, which is associated with increases in gene copy number [13].

ORAI1 has been assessed in a variety of cancer models since its identification in 2006 [14], and this has been comprehensively reviewed by other authors [15,16,17,18,19]. In contrast, fewer studies have assessed its related isoform ORAI3 in cancer. ORAI3 is present only in mammals and is described as the “exceptional” ORAI isoform [20]. Indeed, the extended length of the ORAI3 extracellular domain may reflect distinct regulatory mechanisms for ORAI3 [20]. ORAI3 is more insensitive to inhibition by oxidation than ORAI1, and ORAI3 silencing increases the sensitivity of human effector T helper lymphocytes to reactive oxygen species (ROS) [21]. The potential importance of ORAI3 in the context of external stress responses is also reflected in the increased expression of *ORAI3* in the lungs of mice after staphylococcal infection, where the reduced sensitivity of ORAI3 to ROS-mediated inhibition may be important in immune responses [22]. Hence, ORAI3 may be of particular significance in the tumor microenvironment where hypoxia can contribute to increased levels of ROS [23,24,25]. Indeed, hypoxia in the tumor microenvironment is linked to the activation of a variety of invasive pathways including epithelial to mesenchymal transition (EMT) [25]. However, there are no previous studies of hypoxia effects of ORAI3 in cancer cells.

Studies assessing ORAI3 have highlighted the potential importance of ORAI3 in specific cancer types. In some prostate cancers, disease progression seems to be associated with a switch from ORAI1-mediated Ca^2+^ influx to Ca^2+^ influx mediated by an ORAI1/ORAI3 heteromeric channel, due to genomic alterations in ORAI3 expression and/or tumor microenvironmental factors [26]. The consequences of this remodeling are increased proliferation and apoptotic resistance [26]. More recently, ORAI3 levels have been related to metastasis and poor survival in lung adenocarcinomas [27]. In the context of breast cancer, ORAI3 silencing has anti-proliferative effects on estrogen receptor-α (ERα)-positive MCF-7 cells in vitro and in vivo [28,29], but no effect on the anchorage-independent growth of ERα-negative/basal/triple negative MDA-MB-231 breast cancer cells [29]. Further evidence of association between ERα status and breast cancer, is the report of increased levels of ORAI3 in ERα-positive breast cancer cell lines compared to ERα-negative breast cancer cell lines, the contribution of ORAI3 to SOCE in ERα-positive breast cancer cell lines but not those which lack the ERα [30] and the ability of ERα silencing to significantly reduce *ORAI3* expression in MCF-7 cells [29]. However, the relationship between ORAI3 levels and breast cancer subtypes has not been extensively evaluated in clinical samples. In this study, we sought to define *ORAI3* mRNA expression in breast cancers of different molecular subtypes and compare expression profiles in relation to *ORAI1* expression. The potential role of increased gene copy number on *ORAI3* and *ORAI1* expression in breast cancer subtypes was also evaluated. The sensitivity of ORAI3 expression to hypoxia was assessed in breast cancer cells. Finally, silencing siRNAs were used to help identify possible pathways that may be regulated by ORAI3 in an ERα-negative basal/TNBC cell line with known hypoxia-driven cellular plasticity.

## 2. Material and Methods

### 2.1. Cell Culture

The MDA-MB-468 cell line was obtained from The Brisbane Breast Bank, UQCCR, Brisbane, QLD, Australia and maintained in Dulbecco’s Modified Eagle’s Medium (DMEM) with high glucose (Sigma-Aldrich, St Louise, MO, USA), supplemented with 4 mM L-glutamine 10% fetal bovine serum (FBS). MDA-MB-468 cells stably expressing the GCaMP6m sensor were maintained in the media described above with the addition of 0.5 µg/mL puromycin (Sigma-Aldrich). The HCC1569 and MDA-MB-231 cell lines were obtained from The American Type Culture Collection (ATCC, Manassas, VA, USA) and cultured in RPMI-1640 media (Sigma-Aldrich) and DMEM respectively, both with 10% FBS. The PMC42LA cell line was obtained from Dr. Leigh Ackland, Deakin University, Melbourne, Australia [31,32], and maintained in RPMI-1640 media with 10% FBS. Cells were maintained in 37 °C and 5% CO_2_ in a humidified incubator. For hypoxia experiments, 24 h post plating cells were serum starved (0.5% FBS) for 24 h and placed in a hypoxic incubator (1% O_2_, 5% CO_2_ and 94% N_2_) for periods stated in the results. For the EGF experiment, 24 h post serum reduction, cells were treated with 50 ng/mL EGF (E9644; Sigma-Aldrich) for 24 h prior to RNA isolation. Cell lines were routinely tested for mycoplasma using MycoAlert kit (Lonza, Basel, Switzerland) and validated by STR profiling using The GenePrint^®^ 10 System (Promega, Madison, WA, USA) at QIMR Berghofer, Brisbane, QLD, Australia.

### 2.2. Real-Time RT-PCR

Total RNA was isolated and purified using a RNeasy Plus Mini Kit (74134; Qiagen). Reverse transcription was performed using the Omniscript RT Kit (205111, Qiagen, Hilden, Germany). The resulting cDNA was amplified using TaqMan Fast Universal PCR Master Mix (4352042; Life Technologies, Carlsbad, CA, USA) and TaqMan gene expression assays in a StepOnePlus Real-Time PCR system (Life Technologies). Relative target mRNA levels were calculated using the comparative C_T_ (ΔΔC_T_) method and results were normalized to the ribosomal 18S sRNA. The specific Taqman real-time assays used in this study are as follow: 18s ribosomal RNA (4319413E), Snail (Hs00195591_m1), Twist (Hs00361186_m1), N-cadherin (Hs00983062_m1), CD24 (Hs02379687_s1), CD44 (Hs01075861_m1), ORAI1 (Hs00385627_m1), ORAI2 (Hs00259863_m1), ORAI3 (Hs00743683_s1), ID1 (Hs03676575_s1), TREM1 (Hs00218624_m1) and PGF (Hs00182176_m1).

### 2.3. Immunoblotting

MDA-MB-468 cells were lysed using protein lysis buffer containing IGEPAL (1% v/v), sodium deoxycholate (0.5% w/v), Tris (50 mM), NaCl (100 mM), and protease and phosphatase inhibitors. Protein concentrations were determined based on the Bradford method using the colorimetric Protein Assay Dye Reagent (500-0006, Bio-Rad, Hercules, CA, USA). Samples were resolved on NuPAGE 4–12% Bis-Tris Protein Gels (Invitrogen, Carlsbad, CA, USA) or Mini-PROTEAN TGX stain-free precast gels (Bio-Rad) and transferred to polyvinylidene difluoride membranes. Proteins were detected using primary antibodies against EGFR (2232, Cell Signaling, Danvers, MA, USA), phospho-EGFR (Tyr1173; 4407, Cell Signaling), vimentin (V6389, Sigma-Aldrich), HIF-1α (610958, BD Bioscience, San Jose, CA, USA) and β-actin (A5441, Sigma-Aldrich) and horseradish peroxidase-conjugated secondary antibodies goat-anti-mouse (170-6516, Bio-Rad) and goat-anti-rabbit (170-6516, BioRad). Primary antibodies were incubated with membranes overnight at 4 °C except for β-actin antibody, which was incubated for 1 h at room temperature. Secondary antibodies were incubated for 1 h at room temperature. Images were obtained using a Bio-Rad VersaDoc or ChemiDoc Imaging System and quantified using Quantity One (version 4.6.7, Bio-Rad) or Image Lab Software (version 5.2.1, Bio-Rad).

### 2.4. siRNA-Mediated Silencing

Cells were seeded at 1 × 10^4^ (for 24 h hypoxia), 6 × 10^3^ (for 48 h hypoxia) and 3.5 × 10^3^ (for 72 h hypoxia) per well in 96-well plates. 24 h post seeding, DharmaFECT4 Transfection Reagent (0.1 µL per well) and Dharmacon ON-TARGETplus SMARTpool siRNAs (Thermo Scientific, Waltham, MA, USA), which are a mixture of four siRNAs providing advantages in both specificity and potency of gene silencing, were used for silencing ORAI3 (L-015896-00), HIF-1α (L-004018-00) and HIF-1β (L-007207-00). Non-targeting siRNA (siNT; D-001810-10) was used as control treatment.

### 2.5. Measurement of Cytosolic Free Ca^2+^

Fluorometric Imaging Plate Reader (FLIPR^TETRA^, Molecular Devices, Sunnyvale, CA, USA) and the PBX no-wash Ca^2+^ Assay Kit (640,175, BD Biosciences) were used to assess cytosolic free Ca^2+^ ([Ca^2+^]_CYT_) in MDA-MB-468 cells. Cells were seeded at a density of 6 × 10^3^ per well in 96-well CellBIND plates (Corning Costar, MA, USA). Post plating (24 h), ORAI3 was siRNA-silenced as described above. Two days after silencing, cells were serum starved (0.5% FBS) for 24 h and subsequently placed in a hypoxia incubator for 48 h. Ca^2+^ measurement during the SOCE was performed as previously described [33,34]. ScreenWorks Software (v2.0.0.27, Molecular Devices) was used to analyse data, and response over baseline was evaluated as a relative measure of [Ca^2+^]_CYT_.

### 2.6. Cell Migration Assay

Cell motility was assessed using collagen matrices and live cell imaging. Collagen coated plates were prepared by adding 50 µL of a collagen mixture comprising 10× PBS (8% *v*/*v*), DMEM (24% *v*/*v*), collagen type I from bovine skin (final concentration 2 mg/mL; C4243, Sigma-Aldrich), adjusted to physiological pH with 1 M NaOH, to 96-well cell culture plates. Plates were then incubated at 37 °C, 5% CO_2_ for 1 h prior to cell seeding at a density of 1000 cells per well. ORAI3 was siRNA-silenced 24 h post seeding as described above but with 0.5 µL per well DharmaFECT4 Transfection Reagent. Cells were serum starved (0.5% FBS) for 48 h after silencing for 24 h, and were then placed on the stage of a JuLi^TM^ Stage Live Cell Imaging System (NanoEnTek Inc. Seoul, South Korea) contained within a hypoxia incubator. Cell motility was assessed by capturing bright field images with 4× objective from the centre of wells every 15 min, starting from 72 h post exposure to hypoxia for a period of 12 h (between 72–84 h in hypoxia). Single cell tracking was performed using ImageJ 1.49q software (NIH, Bethesda, MD, USA, website: https://imagej.nih.gov/ij/ (accessed on 10 March 2016) and cell movement was calculated and illustrated with Chemotaxis and Migration Tool V2.0 (Ibidi, Munich, Germany). Strict criteria were applied for the exclusion of cells that were dying, dividing or moving out of the field of view during the period of assessment.

### 2.7. Development of MDA-MB-468-GCaMP6m Cell Line

GCaMP6m was amplified from pGP-CMV-GCaMP6m (a gift from Dr. Douglas Kim, Addgene plasmid #40754) and cloned with BamHI and NotI restriction enzymes into pCDH-EF1-FHC lentiviral vector (a gift from Dr. Richard Wood, Addgene plasmid #64874). The sequence of GCaMP6m was fully verified by Sanger sequencing. Lentiviral vectors pCMV-VSV-G, pCMV-dR8.2 dvpr (a gift from Dr. Bob Weinberg, Addgene plasmids #8454 and #8455) and pCDH-EF1-FHC-GCaMP6m were transfected into HEK293T cells using Lipofectamine 3000 (Thermo Scientific, Waltham, MA, USA) as per the manufacturer’s protocol. Post-transfection (48 h) the viral supernatant was collected and used to transduce MDA-MB-468 cells, in the presence of 8 µg/mL polybrene (Merck, Darmstadt, Germany). After 48 h, cells were treated with 1 µg/mL of puromycin (Sigma-Aldrich). Transduced MDA-MB-468-GCaMP6m cells were sorted into single colonies via a Beckman Coulter MoFlo Astrios EQ, expanded, and validated as previously described [35].

### 2.8. Assessment of Spontaneous Ca^2+^ Transients

MDA-MB-468-GCaMP6m cells were seeded at a density of 6 × 10^3^ cells per well in 96-well black-walled imaging plates (Corning Falcon) and underwent ORAI3 silencing and hypoxic treatment as described above. Following hypoxic exposure, media was changed to physiological salt solution (PSS, 5.9 mM KCl, 1.4 mM MgCl_2_, 10 mM HEPES, 1.2 mM NaH_2_PO_4_, 5 mM NaHCO_3_, 140 mM NaCl, 11.5 mM glucose, 1.8 mM CaCl_2_, pH 7.3) and cells were imaged every 2 s for 60 s using a 10× objective and an automated epi-fluorescent microscope ImageXpress Micro (Molecular Devices). Excitation and emission wavelengths were 472/30 nm and 530/35 nm, respectively. Automated image segmentation using MetaXpress Software (v. 6.2.3.733, Molecular Devices), was conducted as previously described [35]. Responses of individual cells were analysed for peaks and troughs in relative GCaMP6m fluorescence intensity, with spontaneous Ca^2+^ activity considered to have occurred if a peak was a minimum of 1.5% greater in relative fluorescence intensity than the preceding trough.

### 2.9. ORAI3 Promoter Analysis

Histone methylation data was sourced from ENCODE through the IGV browser [36,37]. Phastcons and CpG islands were also accessed through the IGV browser from the data registry of the Broad Institute [37]. ChIP-Seq data for HIF1α and HIF1β was sourced through the Gene Expression Omnibus (GSE59937 [38]). The HIF motif was published as RCGTGM [39]. The IGV snapshot was taken on the combined data using IGV for Mac Desktop v2.3.81 [37]. 

### 2.10. Analysis of ORAI1 and ORAI3 Expression in Breast Tumors

RNA-Seq data for the TCGA dataset [40] was sourced cBioPortal for Cancer Genomics [41,42] and includes 1100 tumors, 140 Basal-like (Basal), 67 HER2-enriched (HER2), 420 Luminal A (LumA), 194 Luminal B (LumB), 24 Normal-Like (N-Like) and 255 not yet classified tumors. RNA-Seq expression data was downloaded as RNA-Seq by expectation maximisation (RSEM [43]), log2 transformed and graphed using Graphpad Prism 7 (v7.0b for Mac). For generation of the heat-map, expression of ORAI1, ORAI3 and the relevant molecular markers was mean-centered and hierarchically clustered in Multiple Experiment Viewer (MeV [43]) via Manhattan-based average-linkage clustering. 

Copy number alterations (CNA) were sourced from TCGA as above but include fewer samples with 838 tumors, 136 Basal-like (Basal), 69 HER2-enriched (HER2), 415 Luminal A (LumA), 194 Luminal B (LumB) and 24 Normal-Like (N-Like). GISTIC [44] CNA were pre-calculated by the TCGA consortium and numbers correspond to: −2 = homozygous deletion, −1 = hemizygous deletion, 0 = neutral/no change, 1 = gain and 2 = high level amplification.

Expression levels for the TNBC subtypes was sourced from R2 Genomics Analysis and Visualization Platform (http://r2.amc.nl). The Brown TNBC breast tumor cohort [7] was used to asses ORAI3 expression within TNBC subtypes. The four subtypes consisted of 198 tumors in total, including 54 Basal-like immune activated (BLIA), 60 Basal-like immune-suppressed (BLIS), 37 Luminal androgen receptor (LAR) and 47 mesenchymal (MES). Graphed are the normalized array probe values. 

### 2.11. Patient Survival Analysis

Analysis of patient survival was carried out using the online Kaplan-Meier Plotter tool [45]. Survival was stratified using the ‘Auto select best cutoff’ and cohorts selected as ERα-negative (ERα-), Basal: ER- and HER2-negative, TNBC: ERα-, HER2 and progesterone receptor negative (PR-). For analysis of ORAI3/ORAI1 ratios stratifying patient survival, ORAI1 was assessed in inverse. Kaplan-Meier Plotter combined the average expression of ORAI3 with the inverse expression for ORAI1, so that a tumor with high ORAI3 must have low ORAI1. This is done with the option “Use multigene classifier,” then the average expression of both probes and the “invert” option on for ORAI1. Expression cut-offs are automatically determined by the tool. Pietenpol TNBC subtypes were defined in Lehmann et al. 2011 [8]. Patient numbers within the high and low expression groups are indicated below each graph. Hazard ratios (HR) with 95% confidence intervals and log-rank *p*-values are indicated on each graph. Affymetrix probes used were, 226531_at: *ORAI1* and 221864_at: *ORAI3*.

MedCalc (version 12.7, Ostend, Belgium) was used to calculate multivariate and univariate Cox proportional hazard regressions based on gene expression and clinical traits. Receiver operator characteristic (ROC) curves were generated for expression of ORAI1, ORAI2, ORAI3 and MKI67 and expression cut-offs for ‘high’ and ‘low’ chosen based on the maximum deviation from the ‘random guess’ line. Lymph node status was designated as negative (−) or positive (+, ≥1 node infiltrated at time of surgery). Tumor grade and stage were sourced from METABRIC [46]. Tumor size categories were as follows T1 ≤ 20mm, T2 ≥ 20 mm and < 50 mm and T3 ≥ 50 mm.

### 2.12. RNA Sequencing Analysis

Library preparation and sequencing were performed by the IMB Sequencing Facility, University of Queensland, St Lucia, QLD, Australia. mRNA integrity was assessed on an Agilent 2100 Bioanalyzer (Agilent Technologies, Palo Alto, CA, USA), and were of high quality (RNA integrity numbers [RIN] of 8.7–9.2). mRNA was converted to sequencing libraries using TruSeq Stranded Total RNA Sample Prep Kit (Illumina™, San Diego, CA, USA), and sequenced on a NextSeq 500 using high output v1 chemistry and single-end 76bp reads, resulting in >32 million reads per replicate. Reads were mapped against the reference genome (UCSC hg19) using STAR [47], with >26 million uniquely mapped reads per replicate. Gene-level counts were generated using htseq-count in the HTSeq python package [48] with the UCSC annotation, and differential expression was detected using the edgeR [49] packages in R. These data have been deposited in Gene Expression Omnibus (accession number GSE107692.

### 2.13. Statistical Analysis

GraphPad Prism Version 7.00 for Windows, GraphPad Software (La Jolla, CA, USA) was used for statistical analysis. Specific statistical tests used for each experiment are described in the corresponding figure legend.

## 3. Results

### 3.1. ORAI1 and ORAI3 in Breast Cancer Molecular Subtypes

**Figure 1:**
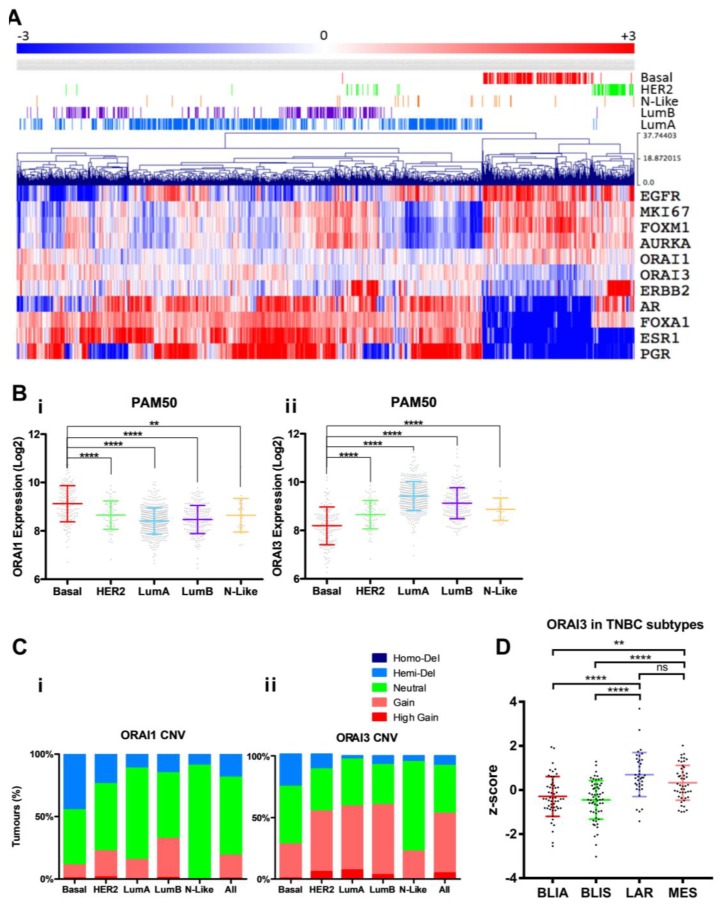
ORAI1 and ORAI3 are differentially expressed and amplified in breast cancer. (**A**) RNA-Seq gene expression for ORAI1, ORAI3 and commonly used molecular markers of breast cancer subtypes. Displayed above the heatmap are the PAM50 molecular subtypes. RNA-Seq data is log2 transformed RSEM and mean-centered, then clustered via Manhattan-based average-linkage. (**B**) RNA expression of ORAI1 (i) and ORAI3 (ii) within each of the PAM50 molecular subtypes. Subtypes were compared to the basal-like tumors and significance assessed via a one-way ANOVA with multiple comparisons (*p*-values are ** ≤0.01, **** ≤ 0.0001). (**C**) GISTIC copy number analysis for ORAI1 (i) and ORAI3 (ii) across the PAM50 molecular subtypes. Shown is the percentage of tumors within each subtype and the CNV. Tumor RNA-Seq and CNV sourced from TCGA. Basal-like (Basal), HER2-enriched (HER2), Luminal A (LumA), Luminal B (LumB) and normal-like (N-Like). (**D**) Expression for *ORAI3* within the TNBC subtypes as determined by Brown et al. [7]; Basal-Like Immune-Suppressed (BLIS), Basal-Like Immune-Activated (BLIA), Luminal-Androgen Receptor (LAR) and Mesenchymal (MES). Data sourced from Brown et al. [7] via R2 Genomics Analysis and Visualization Platform. ns = not significant (*p* ≥ 0.05), ** *p* < 0.01, **** *p* < 0.0001, (one-way ANOVA, with Tukey’s multiple comparisons).

**Figure 2:**
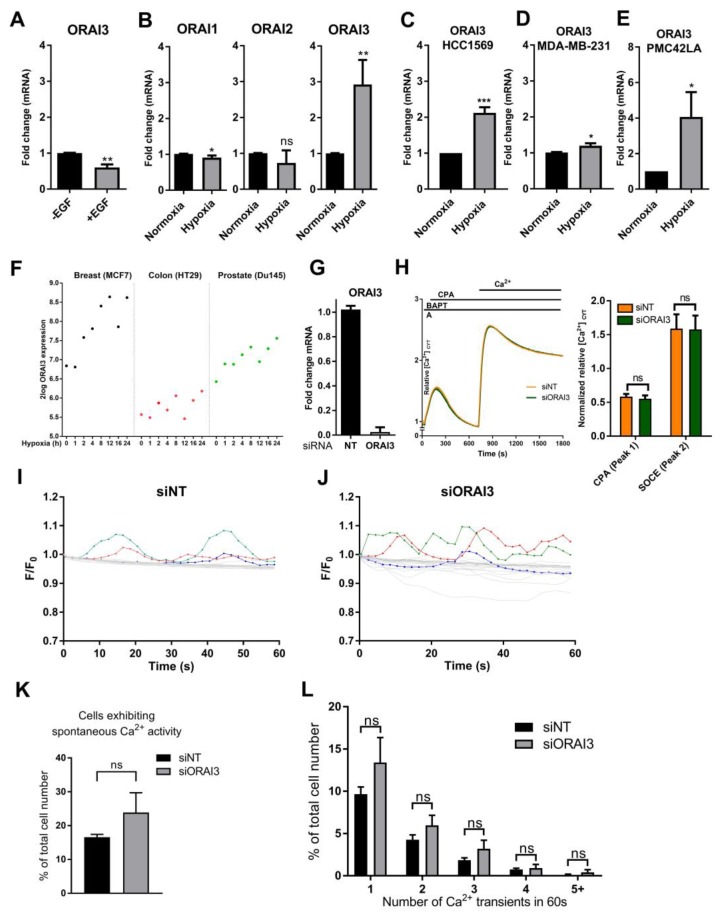
Hypoxia induces *ORAI3* expression. (**A**) *ORAI3* expression after EGF treatment (24 h, 50 ng/mL) treatment in MDA-MB-468 cells (**B**) mRNA expression of *ORAI1*, *ORAI2* and *ORAI3* after hypoxia (24 h, 1% O_2_) in MDA-MB-468 breast cancer cells compared to cells maintained in normoxia. (**C**) Induction of *ORAI3* expression after hypoxia (24 h) in HCC1569, (**D**) MDA-MB-231 and (**E**) PMC42LA breast cancer cells. (**F**) Assessment of *ORAI3* expression in breast (MCF7), colon (HT29) and prostate (Du145) after exposure of cells to normoxia (time-point 0 h) and different times of severe hypoxia (0% O_2_ for 1, 2, 4, 8, 12, 16 and 24 h, respectively) extracted from the publicly available data [47] using the R2 genomics analysis platform. (**G**) confirmation of siRNA-mediated Orai3 silencing and (**H**) FLIPR traces (left) and quantification (right) of the mean [Ca^2+^]_CYT_ levels during store-operated Ca^2+^ entry with ORAI3 silencing (siORAI3) and non-targeting siRNA control (siNT) in MDA-MB-468 cells exposed to 48 h hypoxia. (**I**) Spontaneous Ca^2+^ transients in MDA-MB-468 cells expressing GCaMP6m following ORAI3 silencing (siORAI3) and 48 h exposure to hypoxia. Ca^2+^ transients in 20 individual cells unbiasedly selected from a single well following silencing of non-targeted siRNA control (**I**), or ORAI3 (**J**). Highlighted lines represent examples of cells exhibiting spontaneous Ca^2+^ activity. (**K**) Percentage of cells exhibiting spontaneous Ca^2+^ activity. (**L**) Stratification of cells by number of Ca^2+^ transients in 60 s. ns = not significant (*p* ≥ 0.05), * *p* < 0.05, ** *p* < 0.01, *** *p* < 0.001 (unpaired t-test), *n* = 3, mean ± SD.

To define levels of ORAI1 and ORAI3 in breast cancer molecular subtypes, we assessed RNA sequencing (RNA-Seq) data from the TCGA breast cancer database (845 tumors). Figure 1A shows breast tumors stratified by expression of molecular markers, clustering PAM50 subtypes. As expected, breast cancers of the basal molecular subtype had low levels of the ER and PR and higher levels of the epidermal growth factor receptor (EGFR). Consistent with reports from microarray data indicating higher levels of ORAI1 in basal breast cancers compared to non-basal [11], our assessment of five intrinsic PAM50 molecular subtypes showed higher levels of *ORAI1* in basal breast cancers compared to HER2-enriched, Luminal A, Luminal B and Normal-like subtypes (Figure 1A), with significantly higher levels of *ORAI1* in basal breast cancers compared to all other molecular subtypes (Figure 1(Bi)). The mechanism for elevated *ORAI1* in basal breast cancers is unlikely to involve increases in *ORAI1* gene copy number, since copy number variances did not follow the same trend as mRNA levels (Figure 1(Ci)). Consistent with the reported lower levels of *ORAI3* in ER-negative breast cancer cell lines, breast cancers with low levels of *ORAI3* were most evident in basal breast cancers (Figure 1A) and basal breast cancers had significantly lower levels of *ORAI3* than the other molecular subtypes (Figure 1(Bii)). Copy number variance analysis indicated that the luminal molecular subtypes (which overlap with ERα-positive breast cancers) had a much higher proportion of breast cancers with ORAI3 gain or high gain than the basal molecular subtype (Figure 2(Cii)) and hence this may be a mechanism for elevated *ORAI3* levels in these subtypes. Given that gains in *ORAI3* gene copy number were also evident in the basal subtype (Figure 1(Cii)), and some basal breast cancers were associated with high levels of *ORAI3* (Figure 1(A,Bii)), we assessed if *ORAI3* levels were elevated in any of the TNBC molecular subtypes. This analysis indicated that *ORAI3* levels were significantly higher in both the MES and LAR triple negative subtypes compared to those of the BLIA and BLIS subtypes (Figure 1D).

### 3.2. Hypoxia Induces ORAI3 Expression

Since *ORAI3* is significantly up-regulated in the TNBC cell MES subtype, compared to BLIS and BLIA triple-negative breast cancer cell subtypes, we assessed the effect of two different epithelial to mesenchymal transition (EMT) inducers on the expression of *ORAI3* in triple negative, basal-like MDA-MB-468 breast cancer cells. The EMT inducer in this model, epidermal growth factor (EGF) [50,51], did not increase *ORAI3* levels (Figure 2A) and instead a significant reduction in the level of *ORAI3* was observed. In contrast, the other EMT inducer assessed, hypoxia [52], significantly elevated levels of *ORAI3*, with no increases in the related isoforms *ORAI1* and *ORAI2* (Figure 2B). *ORAI3* levels increased in two other basal-like breast cancer cell lines (HCC1569 and MDA-MB-231) and the EMT breast cancer cell line model PMC42LA, under hypoxic conditions (Figure 2C–E). Assessment of a publicly available microarray dataset [53], showed that increases in *ORAI3* were also evident in ERα-positive MCF-7 breast cancer cells, HT29 colon cancer cells and Du145 prostate cancer cells proportionate to increased exposure to hypoxia (Figure 2F). In contrast, *ORAI1* and *ORAI2* were not consistently up-regulated with hypoxia in these cell lines (Figure S1). Given the reports of ORAI3 contribution to SOCE in ERα-positive breast cancer cell lines with high levels of ORAI3 [30], we assessed whether ORAI3 contributed to SOCE in MDA-MB-468 cells after its induction by hypoxia. ORAI3 silencing (Figure 2G) did not reduce SOCE in MDA-MB-468 cells after hypoxia (Figure 2H), indicating that ORAI3 did not function as a SOCE mechanism in this model of hypoxia. To assess possible subtle effects on [Ca^2+^]_CYT_ signals, the effect of ORAI3 silencing during hypoxia on spontaneous [Ca^2+^]_CYT_ oscillations, as previously described in MDA-MB-468 breast cancer cells [54], was also evaluated. No significant effect on this parameter was observed (Figure 2I–L), suggesting that ORAI3 may regulate highly localized Ca^2+^ changes and/or [Ca^2+^]_CYT_ only at specific times of hypoxia that could not be assessed in our studies.

### 3.3. HIF1α Controls the Hypoxic Expression of ORAI3

**Figure 3:**
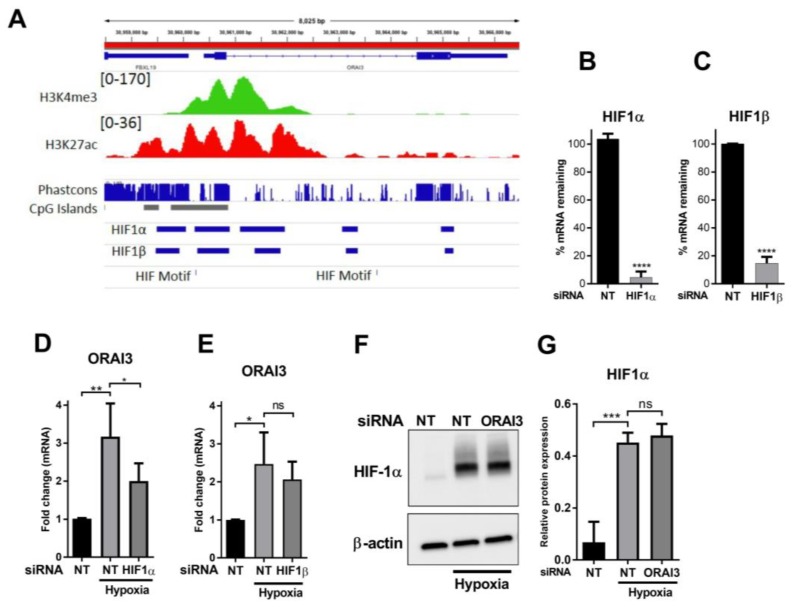
Hypoxia-induced *Orai3* up-regulation is HIF1α dependent. (**A**) The *ORAI3* genomic region on human chromosome 16. Displayed are the histone modifications (human m epithelial cells, HMEC) indicative of active promoters, conservation (Phastcons), CpG islands, HIF1 binding (MCF7 cells) and HIF motifs. Confirmation of HIF1α (**B**) and HIF1β (**C**) siRNA-mediated silencing and the effect of HIF1α (**D**) and HIF1β (**E**) silencing (**** *p* < 0.0001, unpaired t-test) on the induction of *ORAI3* by hypoxia in MDA-MB-468 cells. (**F**) Representative immunoblot and (**G**) densitometry analysis of the effect of *ORAI3* silencing on hypoxia-mediated increases of HIF1α protein in MDA-MB-468 cells compared to NT siRNA in normoxia and hypoxia. ns = not significant (*p* ≥ 0.05), * *p* < 0.05, ** *p* < 0.01, *** *p* < 0.001 (one-way ANOVA, with Bonferroni’s multiple comparisons), *n* = 3, mean ± SD.

**Figure 4:**
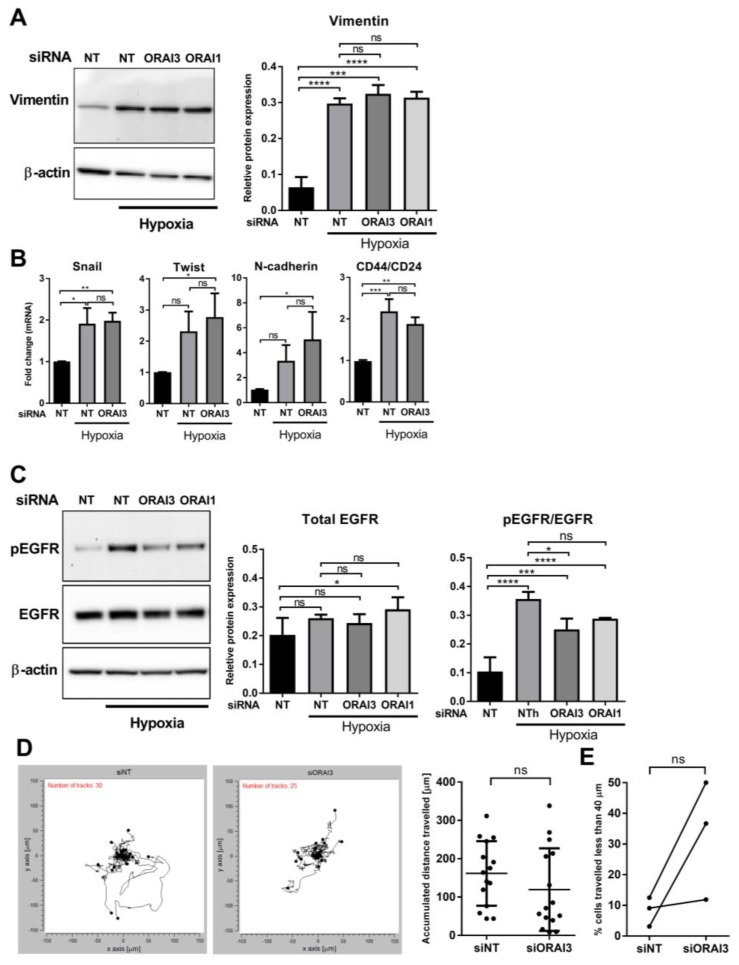
Effect of *ORAI3* silencing on the levels of hypoxia-induced expression of EMT markers, EGFR activation and cell migration of MDA-MB-468 breast cancer cells. (**A**) Representative immunoblot (left) and densitometry analysis (right) of the effect of ORAI3 and ORAI1 silencing on hypoxia-induced vimentin protein expression and (**B**) the effect of ORAI3 silencing on the mRNA expression of key hypoxia-induced EMT markers in MDA-MB-468 breast cancer cells. (**C**) Representative immunoblot (left) and densitometry analysis (right) of the effect of ORAI3 and ORAI1 silencing on the total levels of EGFR protein and hypoxia-induced EGFR phosphorylation. ns = not significant (*p* ≥ 0.05), * *p* < 0.05, ** *p* < 0.01, *** *p* < 0.001, **** *p* < 0.0001, (one-way ANOVA, with Tukey’s multiple comparisons), *n* = 3, mean ± SD. (**D**) Representative spatial plot of all NT control- and ORAI3-silenced cells from one well of the same experiment (left) and quantitative analysis of the distance travelled by 15 randomly selected cells from three independent experiments (5 cells from each experiment). (**E**) Quantitative analysis of the percent of cells in each experiment from three independent experiments that travelled less than 40 µm. Total number of cells from three independent experiments analyzed were 95 and 80 cells for siNT and siORAI3, respectively. Cell migration was analysed in hypoxic conditions over a period of 12 h after exposing cells to 72 h hypoxia. ns = not significant (*p* ≥ 0.05) (Mann-Whitney *U*-test), *n* = 3, mean ± SD.

The potential mechanism for *ORAI3* up-regulation with hypoxia was first assessed via analysis of the promotor region of the *ORAI3* gene. We defined the *ORAI3* promoter region by the presence of CpG islands, high conservation and enrichment of H3K4me3 and H3K27ac in normal human mammary epithelial cells (HMECs). Binding motifs for the hypoxia response regulators and transcription factors HIF1α and HIF1β were present in the *ORAI3* promotor in MCF7 cells indicating an ability for HIF1 factors to bind in breast cancer cells (Figure 3A). Silencing of HIF1α or HIF1β (Figure 3B,C), demonstrated that hypoxia-mediated *ORAI3* induction was mediated by HIF1α and not HIF1β (Figure 3D,E). However, ORAI3 silencing suggested that ORAI3 is not a regulator of HIF1α after hypoxic exposure in MDA-MB-468 Basal-A triple negative breast cancer cells (Figure 3F,G). Given the induction of *ORAI3* by hypoxia and the up-regulation of *ORAI3* in the MES TNBC molecular subtype, we assessed the effects of ORAI3 silencing on the induction of EMT markers by hypoxia. Hypoxia produced robust induction of vimentin protein expression, but these changes were insensitive to ORAI3 silencing (Figure 4A). Similarly, ORAI3 silencing had no significant effect on hypoxia-mediated changes in mRNA levels of the EMT markers Snail, Twist, N-Cadherin, CD44/CD24 ratio (Figure 4B). Combined with the inability of another EMT inducer EGF to promote *ORAI3* expression, these results suggest that ORAI3 is not a ubiquitous regulator of EMT. We therefore assessed another consequence of hypoxia in this model, phosphorylation of EGFR [53]. ORAI3 but not ORAI1 silencing significantly reduced hypoxia-induced increases in EGFR phosphorylation, although ORAI1 silencing was close to significance (*p* value of 0.0546) (Figure 4C). Due to the potential role for EGFR activation on cell migration [55,56], we assessed the consequences of ORAI3 silencing on the migration of MDA-MB-468 cells during hypoxia using collagen matrices and live cell imaging. ORAI3 silencing had no significant effect on the migration of MDA-MB-468 breast cancer cells under these hypoxic conditions (Figure 4D,E), although there was a trend for an increased proportion of cells with low levels of migration (Figure 4E).

### 3.4. ORAI3 Is Involved in the Hypoxic Regulation of Genes Associated with Cell Migration and Inflammatory/Immune Responses

**Figure 5:**
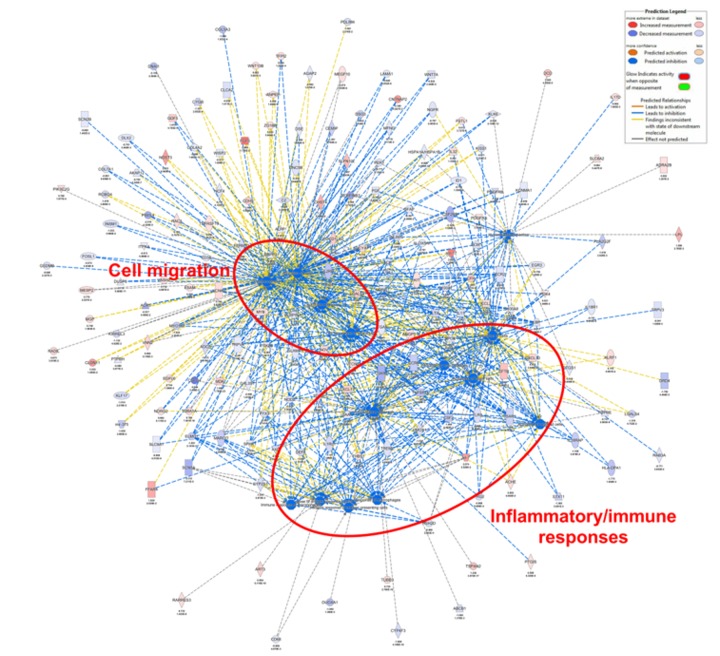
Next generation sequencing data suggesting ORAI3 silencing reduces the expression of genes associated with cell migratory and inflammatory/immune responses. Networks of genes associated with biological functions (nodes) that were significantly altered with ORAI3 siRNA-mediated silencing after hypoxia (1% O_2_, 72 h), identified using Ingenuity Pathways Analysis. Suppressed biological functions are represented in blue nodes. Only biological functions with a z-score below −2.3 are shown (refer to Table S1). No processes were augmented. Biological functions related to a same category were manually circled and labelled with cell migration or inflammatory/immune responses.

**Figure 6:**
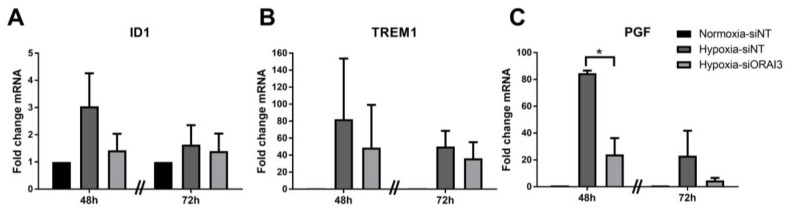
Effect of ORAI3 silencing on the expression of three selected genes from RNA-Seq data that were also up-regulated by hypoxia. Real time RT PCR assessment of the expression of three ORAI3-regulated targets from RNA-Seq that their expression was also up-regulated with hypoxia; (**A**) ID1, (**B**) TREM1 and (**C**) PGF. * *p* < 0.0001 (one-way ANOVA, with Bonferroni’s multiple comparisons), *n* = 3, mean ± SD.

To assess the potential role of ORAI3 on responses to hypoxia, RNA-Seq was conducted comparing gene expression under conditions of hypoxia with and without ORAI3 silencing. Gene ontology of differentially expressed genes demonstrated that ORAI3 silencing led to the alterations in the expression of genes affecting migration and inflammatory/immune responses (Figure 5). There was a significant decrease in the activity of 21 annotated functions with ORAI3 silencing (most of which related to cell migration and inflammatory/immune responses); In contrast there were no functions that increased with ORAI3 silencing during hypoxia (Table S1). Using real time RT-PCR, we further assessed specific targets from the gene sets associated with the migration and immune pathways that were reduced by ORAI3 silencing at both the 48 h and 72 h hypoxia time-points. Three of these assessed genes were significantly induced by hypoxia; Inhibitor of Differentiation 1 (ID1), Triggering Receptor Expressed on Myeloid cells 1 (TREM-1) and Placental Growth Factor (PGF) (Figure 6). At 48 h, there was a trend for ORAI3 silencing to attenuate induction of *ID1* and *TREM-1* and ORAI3 silencing significantly reduced hypoxic induction of *PGF*. These data support the general conclusion that ORAI3 silencing may remodel the expression of genes involved in migration and immune pathways.

**Figure 7:**
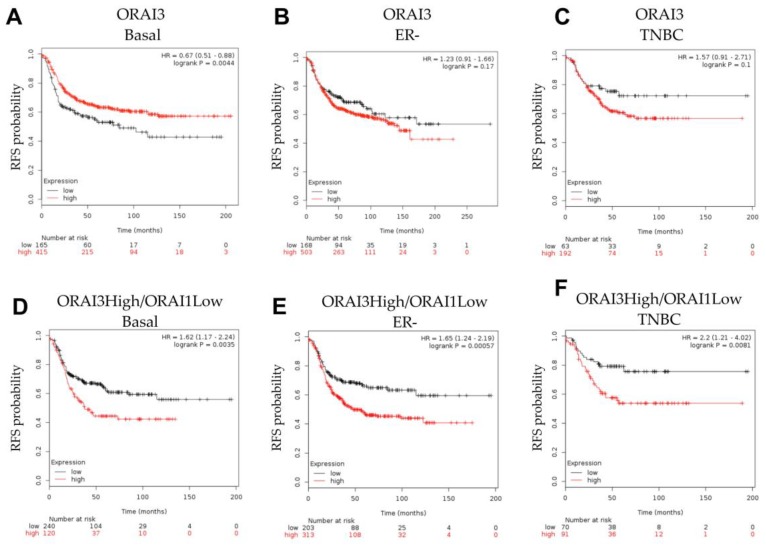
Expression of *ORAI3* in combination with *ORAI1* stratifies the survival of Basal, ER- and TNBC. Stratification of patient relapse free survival (RFS) in basal, estrogen-receptor negative (ER-) and triple negative breast cancer (TNBC) from the KM Plotter cohorts. Stratification of patient survival was based on *ORAI3* expression in (**A**–**C**), and on *ORAI3* in combination with *ORAI1* expression in (**D**–**F**), where ‘high’ group (red) is high expression of *ORAI3* and low expression of *ORAI1* and the ‘low’ group (black) is low expression of *ORAI3* and high expression of *ORAI1*.

To further explore the role of ORAI3 in the context of basal breast cancers, patient survival was stratified based on *ORAI3* gene expression using KM Plotter cohorts. *ORAI3* levels were not an indicator of poorer prognosis as assessed by relapse free survival (RFS) in basal breast cancers (Figure 7A), instead ORAI3 levels were actually associated with improved RFS. *ORAI3* levels were not associated with RFS in ERα-negative and TNBC breast cancers (which have a significant overlap with the basal molecular subtype [5]; Figure 7B,C). Given the reported importance of the balance between *ORAI3*/*ORAI1*, in the context of a more proliferative phenotype in prostate cancer cells [26], and our identification that some basal breast cancers had high *ORAI3* but low *ORAI1* levels (Figure 1A), we compared RFS in breast cancers with high *ORAI3*/low *ORAI1* with other breast cancers. Breast cancers with high *ORAI3*/low *ORAI1* were associated with poorer RFS in basal, ERα-negative and TNBCs, and low ORAI3/high ORAI1 associated with good outcome (Figure 7D–F). Assessment of the TNBC subtypes [8], identified that high *ORAI3*/low *ORAI1* was also associated with significantly poorer RFS, in the basal like-1, mesenchymal and mesenchymal stem-like TNBC subtypes (Figure S2), but no significant relationship was observed in other TNBC subtypes (Figure S2). Our analysis also showed that within basal and ER-breast cancers, higher levels of *ORAI1* were associated with better relapse free survival in patients (Figure S3). In contrast to the *ORAI1/ORAI3* ratio in TNBC (Figure 7F), *ORAI1* levels were not significantly associated with patient RFS in TNBC (Figure S3).

Given the significant association between *ORAI3* and patient prognosis we tested its utility against commonly used clinical markers of patient outcome (Table S2). In this analysis and cohort, high levels of *ORAI3* alone rather than a high *ORAI3*/low *ORAI1* was linked to a to poorer prognosis as was *ORAI2*. In this analysis there was no association between *ORAI1* and prognosis.

## 4. Discussion

Alterations in Ca^2+^ influx via ORAI channels and/or the remodeling of their expression has been identified in several cancers including breast, prostate, lung and colon [26,57,58,59]. The nature and consequences of this remodeling can differ between cancer types. For example, in some prostate cancer cells, reduced ORAI1-mediated Ca^2+^ influx occurs and may bestow resistance to some apoptotic pathways [26], whereas in some breast cancer cells, augmentation of ORAI1-mediated Ca^2+^ influx appears to contribute to migration and proliferation pathways [60]. Although ORAI1 silencing studies have indicated that ORAI1 is a contributor to the proliferation and invasiveness of both basal and non-basal breast cancer cells in vitro, reports of increased *ORAI1* mRNA levels in basal compared to non-basal breast cancers suggested that ORAI1 may make a greater contribution to these functions in breast cancer subtypes [11]. Our analysis of clinical samples from the TCGA database shows that *ORAI1* levels are significantly higher in patients with basal breast cancers compared to all other breast cancer molecular subtypes. However, this does not seem related to increases in *ORAI1* gene region, as ORAI1 gene copy number was higher in the luminal and HER2 molecular subtypes compared to those of the basal molecular subtype. Hence, further studies are required to define the mechanism by which *ORAI1* levels are elevated in basal breast cancers.

In contrast to *ORAI1*, the enrichment of *ORAI3* in clinical breast cancer samples is largely unexplored. However, detailed in vitro studies using human breast cancer cell lines with different ERα expression status show that *ORAI3* expression is ERα sensitive and that *ORAI3* levels are greater in ERα-positive breast cancer cell lines compared to those that are ERα-negative [29,30]. These cell line-based studies predict that *ORAI3* levels should be low in breast cancers of the basal molecular subtype, due to their extensive overlap with breast cancers that are ERα-negative [4,5]. Indeed, our data showed that *ORAI3* levels are significantly lower in basal breast cancers compared to all other molecular subtypes. Our analysis also suggests that in addition to the previously defined potential for the ERα to contribute to *ORAI3* expression in breast cancer cells, overexpression of *ORAI3* in some breast cancers may be a consequence of increased gene copy number. Indeed, over 50% of breast cancers of the luminal A subtype had a gain or high gain in *ORAI3* gene copy number. Hence, there may be multiple drivers for the up-regulation of *ORAI3* in ERα-positive breast cancers, which include the ERα itself and increased copies of the *ORAI3* gene region.

As discussed above, *ORAI3* levels were significantly lower in basal breast cancers overall; however, *ORAI3* levels were high in some basal breast cancers, and we observed significantly higher *ORAI3* levels in TNBCs of the MES and LAR molecular subtypes. These data collectively suggested that *ORAI3* levels may be inducible under certain conditions in cancer cells. Indeed, our studies show that *ORAI3* is induced in a variety of cancer models by hypoxia. In the case of MDA-MB-468 breast cancer cells, hypoxia-induced induction of *ORAI3* is mediated by HIF1α, placing ORAI3 alongside TRPC1 as a HIF1α-inducible ion channel in this model [61,62]. However, in contrast to TRPC1, ORAI3 silencing did not suppress HIF1α induction by hypoxia, demonstrating that regulation is not bidirectional in the context of hypoxia, HIF1α and ORAI3. Despite the increases in hypoxia-induced *ORAI3* expression in MDA-MB-468 ERα-negative breast cancer cells, this up-regulation of *ORAI3* did not bestow an ability to contribute to SOCE, which is apparent in ERα-positive MCF-7 breast cancer cells where ORAI3 silencing suppresses SOCE [28,29]. We also did not observe any ORAI3 siRNA sensitive increases in [Ca^2+^]_CYT_ induced by arachidonic acid in MDA-MB-468 cells after hypoxia (data not shown), suggesting that the increased *ORAI3* levels did not promote an arachidonic acid-sensitive ORAI1/ORAI3 heteromeric channel phenotype. Consistent with the absence of major hypoxia-induced changes in the nature of Ca^2+^ signaling mediated by ORAI3, and the insensitivity of HIF1α induction to ORAI3 silencing, the effects of ORAI3 silencing on changes induced by hypoxia were subtle. Our data demonstrate ORAI3 is not a global regulator of responses to hypoxia. However, the ability of ORAI3 but not ORAI1 silencing to attenuate EGFR phosphorylation induced by hypoxia suggested that specific events in cancer cells activated by hypoxia are fine-tuned by ORAI3. Whether a direct physical interaction occurs between ORAI3 and EGFR to modulate EGFR phosphorylation and/or a localized gradient of calcium via ORAI3 mediated Ca^2+^ influx contributes to autophosphorylation should be the focus of future studies. Such studies would help define the role of ORAI3 in breast cancer cells that express EGFR.

Our RNA-Seq experiments assessing the consequences of ORAI3 silencing on alterations in gene expression induced by hypoxia in MDA-MB-468 basal breast cancer cells further identified specific functions that may be regulated by ORAI3 in basal breast cancers. Gene ontology classified these changes as the suppression of processes related to cell migration and inflammatory/immune responses. However, ORAI3 silencing did not suppress single cell migration during hypoxia in our cell model. This lack of correlation may be evidence that ORAI3 is not a driver of cell migration in basal breast cancers, but rather is a fine-tuner of cancer cell migration in some in vivo contexts, through changing the suite of expressed genes in response to specific microenvironmental factors. These ORAI3-sensitive genes may play important roles as breast cancer cells escape the hypoxic primary tumor microenvironment. Future studies should also assess the potential opportunities presented by ORAI3 silencing/inhibition to alter inflammatory and immune responses in breast cancer. The important role of ORAI3 in the regulation of the transcription of genes up-regulated by hypoxia (despite its inability to attenuate HIF1α induction) is evidenced by the pronounced suppression of placental growth factor (*PGF*) induction by hypoxia. PGF is a member of the vascular epidermal growth factor (VEGF) family, with identified roles in tumor angiogenesis and inflammatory pathways [63]. It is specifically interesting to note (given our identification of ORAI3 regulation of both migratory and immune/inflammatory pathways by hypoxia) that PGF is believed to reprogram the tumor immune microenvironment and contribute to obesity-promoted breast cancer progression [64]. Our identification of the regulation of *PGF* by ORAI3 during hypoxia was somewhat surprising, given reports that in MDA-MB-231 basal breast cancer cells, hypoxic induction of *PGF* is HIF-dependent [65]; however, *PGF* induction by hypoxia in this investigation was suppressed by simultaneous silencing of both HIF1α and HIF2α and hence this is an area for further study. Furthermore, whether the regulation of EGFR in hypoxia by ORAI3 controls the expression of specific genes, as is the case for TRPC1 in this cell line [61], could be the focus of future studies. Our observation of poorer RFS in some breast cancer subtypes with high *ORAI3* and low *ORAI1* levels, is reflective of the association between the ORAI1 activators STIM1/STIM2 in basal breast cancers, where high *STIM1*/low *STIM2* was associated with poorer survival [11]. The potential importance of the ORAI3/ORAI1 heteromeric channel should now be explored in basal breast cancers and TNBCs, as has been assessed in prostate cancer [26].

## 5. Conclusions

These studies have defined the remodeling of *ORAI1* and *ORAI3* in breast cancer molecular subtypes. Elevated *ORAI1* is a feature of basal breast cancers that are also defined by lower levels of *ORAI3*. However, *ORAI3* is inducible by hypoxia in basal breast cancer cells and other cancer cell lines. In contrast to its major role in the proliferative and invasive properties of ERα-positive breast cancer cell lines, the contribution of ORAI3 to ERα-negative breast cancer cell lines is more specific. ORAI3 appears to be a modulator of specific processes involved in the regulation of cell migration and inflammatory/immune pathways in response to hypoxia.

## Supplementary Materials

The following are available online at https://www.mdpi.com/2072-6694/11/2/208/s1, Figure S1: Assessment of ORAI1 and ORAI2 expression in breast (MCF7), colon (HT29) and prostate (Du145) cancer cells after exposure to normoxia (time-point 0 h) or different times of severe hypoxia (0% O2 for 1, 2, 4, 8, 12, 16 and 24 h, respectively), extracted from publicly available data (Starmans et al., 2012) using the R2 genomics analysis platform (http://r2.amc.nl), Figure S2: The combination of ORAI3 and ORAI1 expression stratifies survival of patients’ relapse free survival (RFS) within the triple negative breast cancers. A-F) Stratification of patient relapse-free survival based on ORAI3 and ORAI1 gene expression. ORAI1 expression was inverted so that the ‘high’ expression group (red) is high expression of ORAI3 and low expression of ORAI1 and the ‘low’ expression group (black) is low expression of ORAI3 and high expression of ORAI1, Figure S3: Stratification of patient relapse free survival (RFS) based on ORAI1 expression in basal, estrogen-receptor negative (ER-) and triple negative breast cancer (TNBC) from the KM Plotter cohorts. Table S1: Biological functions that were significantly predicted to be altered by ORAI3 silencing, Table S2: Univariate and multivariate analysis of Basal tumours from the METABRIC cohort.
