# Supplementary material for: ORAI1 and ORAI3 in Breast Cancer Molecular Subtypes and the Identification of ORAI3 as a Hypoxia Sensitive Gene and a Regulator of Hypoxia Responses
Source: Cancers (Basel). 2019 Feb 11;11(2):208. doi: 10.3390/cancers11020208 (PMC6406924; doi:10.3390/cancers11020208)
Supplement: Supplementary file 1 [file cancers-11-00208-s001.pdf]

Supplementary Material: ORAI1 and ORAI3 in Breast Cancer Molecular Subtypes and the Identification of ORAI3 as a Hypoxia Sensitive Gene and a Regulator of Hypoxia Responses

Iman Azimi, Michael J.G. Milevskiy, Silke B. Chalmers, Kunsala T.D.S Yapa, Mélanie Robitaille, Christopher Henry, Gregory J. Baillie, Erik W. Thompson, Sarah J. Roberts-Thomson and Gregory R. Monteith

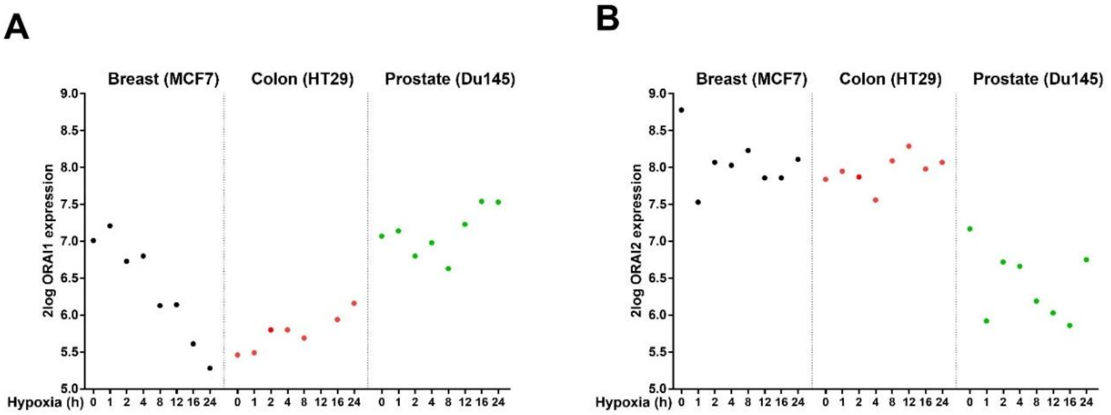

Figure S1. Assessment of *ORAI1* and *ORAI2* expression in breast (MCF7), colon (HT29) and prostate (Du145) cancer cells after exposure to normoxia (time-point 0 h) or different times of severe hypoxia (0% O_2 for 1, 2, 4, 8, 12, 16 and 24 h, respectively), extracted from publicly available data (Starmans et al., 2012) using the R2 genomics analysis platform (http://r2.amc.nl).

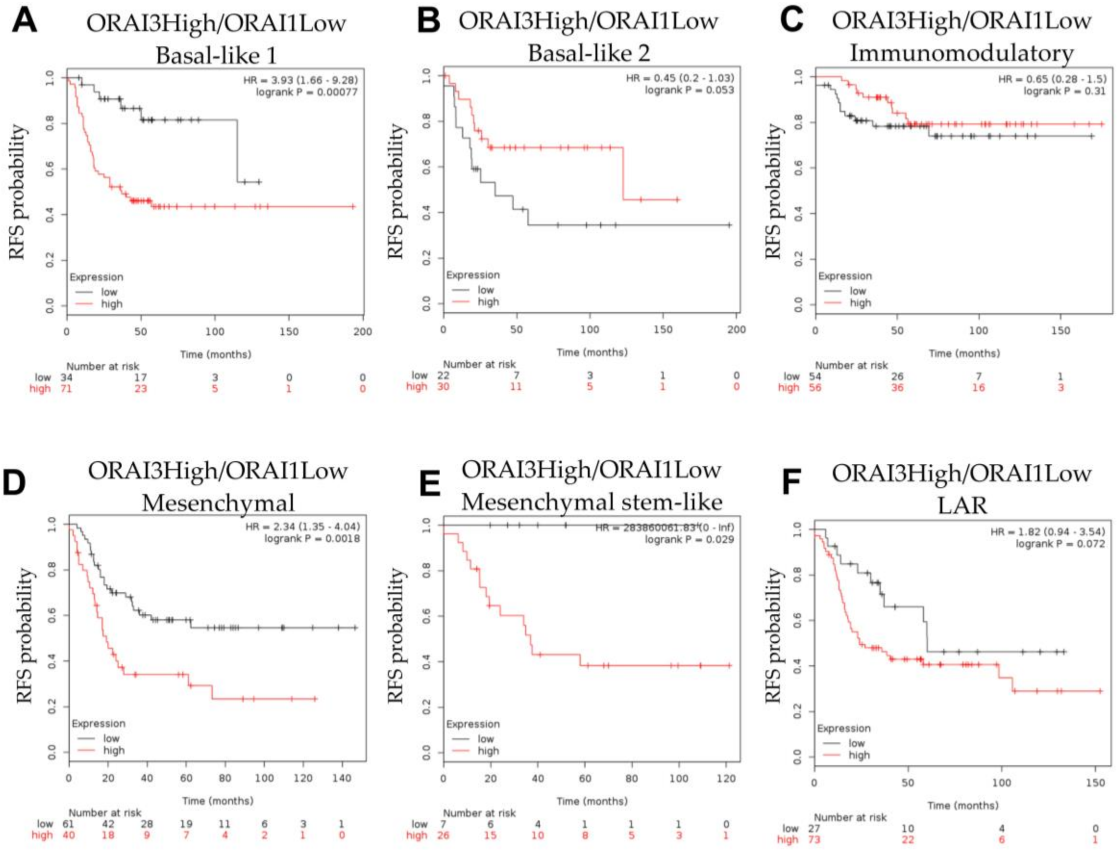

Figure S2. The combination of *ORAI3* and *ORAI1* expression stratifies survival of patients' relapse free survival (RFS) within the triple negative breast cancers. (**A**–**F**) Stratification of patient relapse-free survival based on *ORAI3* and *ORAI1* gene expression. ORAI1 expression was inverted so that the 'high' expression group (red) is high expression of *ORAI3* and low expression of *ORAI1* and the 'low' expression group (black) is low expression of *ORAI3* and high expression of *ORAI1*.

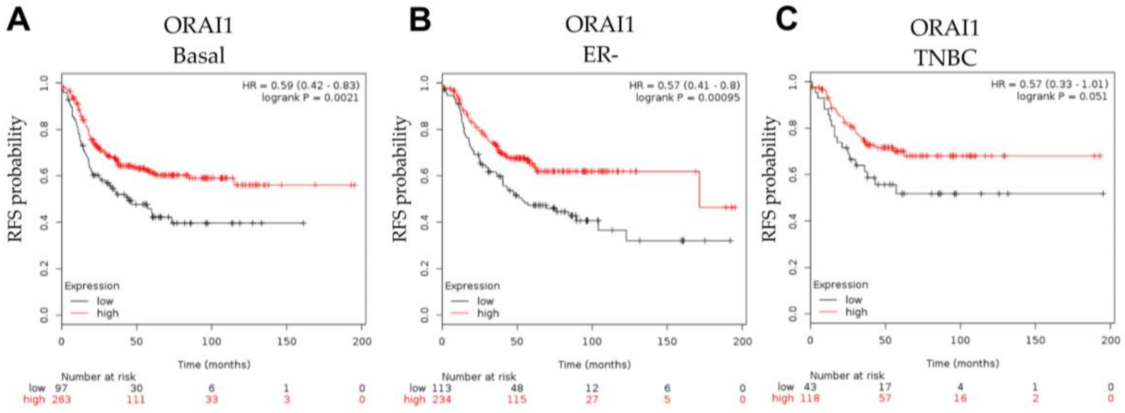

Figure S3. Stratification of patient relapse free survival (RFS) based on *ORAI1* expression in basal, estrogen-receptor negative (ER-) and triple negative breast cancer (TNBC) from the KM Plotter cohorts.

Table S1. Biological functions that were significantly predicted to be altered by ORAI3 silencing.

**Function Annotation**	***p*-Value**	**Predicted Activation**	**Activation *z*-Score**	**# Molecules**
Migration of cells	1.20×10^{-8}	Decreased	−3.219	132
Hypersensitive reaction	9.96×10^{-5}	Decreased	−3.037	33
Cell movement	1.20×10^{-7}	Decreased	−3.022	141
Activation of cells	1.41×10^{-5}	Decreased	−2.946	67
Inflammatory response	3.68×10^{-7}	Decreased	−2.736	62
Leukocyte migration	1.99×10^{-7}	Decreased	−2.636	69
Immune response of antigen presenting cells	9.31×10^{-7}	Decreased	−2.553	22
Response to macrophages	1.51×10^{-6}	Decreased	−2.505	20
Activation of myeloid cells	3.74×10^{-6}	Decreased	−2.349	31
Immune response of macrophages	2.05×10^{-6}	Decreased	−2.345	19
Cell movement of leukocytes	1.88×10^{-6}	Decreased	−2.338	60
Response of antigen presenting cells	2.54×10^{-7}	Decreased	−2.322	24
Infiltration by macrophages	7.74×10^{-6}	Decreased	−2.314	19
Activation of blood cells	6.73×10^{-5}	Decreased	−2.308	51
Engulfment by macrophages	1.65×10^{-6}	Decreased	−2.292	17
Activation of leukocytes	2.12×10^{-5}	Decreased	−2.247	50
Cellular infiltration by macrophages	1.33×10^{-5}	Decreased	−2.154	18
Overweight disorder	1.52×10^{-5}	Decreased	−2.136	41
Obesity	3.06×10^{-5}	Decreased	−2.136	40
Phagocytosis by macrophages	4.76×10^{-6}	Decreased	−2.120	16
Phagocytosis	5.66×10^{-6}	Decreased	−2.046	29

Table S2. Univariate and multivariate analysis of Basal tumours from the METABRIC cohort.

**Condition**	**Univariate Cox-Proportional Hazards Model**			**Multivariate Cox-Proportional Hazards Model (Stepwise)**		
	**HR**	**(95% CI)**	***p*-Value**	**HR**	**(95% CI)**	***p*-Value**
**Age (<40 vs. 41–60 and >60)**	**2.424**	**1.333–4.408**	**0.0385**	**3.013**	**1.607–5.65**	**0.0006**
**Tumour Stage (0 to 4)**	**2.153**	**1.302–3.559**	**0.0029**	**2.298**	**1.382–3.823**	**0.0014**
**ORAI3 (high vs low)**	**1.972**	**1.07–3.636**	**0.0305**	**1.866**	**1.007–3.46**	**0.0485**
**Lymph Node (+ vs. -)**	**2.099**	**1.179–3.738**	**0.0122**	ns		ns
**ORAI2 (high vs. low)**	ns		ns	**3.929**	**1.198–12.94**	**0.0251**
Menopausal Status (Post vs. Pre)	ns		ns	ns		ns
MKI67 (high vs. low)	ns		ns	ns		ns
ORAI1 (high vs. low)	ns		ns	ns		ns
Size (T1, T2, T3)	ns		ns	ns		ns
Tumour Grade (1,2,3)	ns		ns	ns		ns

© 2019 by the authors. Licensee MDPI, Basel, Switzerland. This article is an open access article distributed under the terms and conditions of the Creative Commons Attribution (CC BY) license (http://creativecommons.org/licenses/by/4.0/).
